# The introduction of the concept of anxiety into China: a thematic historical study

**DOI:** 10.3389/fpsyg.2024.1410748

**Published:** 2024-10-28

**Authors:** Peng Miao

**Affiliations:** College of Foreign Languages, University of Shanghai for Science and Technology, Shanghai, China

**Keywords:** anxiety, history, introduction, Traditional Chinese Medicine, xin feng

## Abstract

**Introduction:**

This article presents a thematic historical study on the introduction of “anxiety” to modern China. Based on seminal research conducted previously by European scholars on histories of psychological and psychiatric concepts, this article reviews the itinerary of “anxiety” to China by using multiple types of textual evidence. Alongside a detailed description of how “anxiety” was translated and introduced, a comparison between the notions of “anxiety” in Traditional Chinese Medicine (TCM) and Western psychoanalytic approaches was made. The processes of how “depression” and “anxiety” were introduced to China were also compared.

**Materials and methods:**

Three types of textual evidence were meticulously analyzed. First, lexicographical works published since the 19^th^ century were examined to trace the initial introduction and observe the standardized translation of “anxiety” in Chinese. Second, newspaper and periodical articles were analyzed to understand how knowledge of “anxiety” was communicated to the general public. Third, the evolution of psychological and psychiatric terminologies was observed through medical books.

**Results:**

It was found that “anxiety” was initially translated into various Chinese terms, with “jiaolyu” eventually emerging as the standardized term after 1949. The textual evidence showed that “anxiety” began to be acknowledged as a disease concept in the early 20^th^ century, though it was not formally recognized as a diagnostic category until the late 20^th^ century. This article also highlights the differences between the Western psychoanalytic view on anxiety and the Chinese folk conception of it.

**Discussion and conclusions:**

It was observed that the introduction processes of depression and anxiety to China were similar, though their conceptual histories in the West showed much difference. The fading of neurasthenia marked a new era of psychiatric development in China, characterized by an enhanced specificity in the classification of mental disorders, which was never observed in the discourse about “xin feng,” a corresponding TCM notion of “anxiety.” The article also underscores the need for further exploration of corresponding TCM concepts of “anxiety” and the assimilation of Western psychiatric concepts in China.

## Introduction

Presently, history of concepts, or “Begriffsgeschichte” in German, as a branch of historical studies, is receiving considerable attention in various disciplines around the globe. It unravels the evolving meaning of a concept throughout multiple eras and explores the different perceptions of the concept by different individuals across different languages and cultures (Kirkby, 2020). The study of conceptual history in Europe has a rich tradition in the fields of psychology and psychiatry. Early in 1980s, Jackson (1986) presented a conceptual history of melancholia and depression over the past 2,500 years, tracing their semantic evolution since the Ancient Greek Times. In 2005, “depression” became “reinvented,” as its history of treatment in primary care was presented by Callahan and Berrios (2005). Berrios, in the past 30 years, has constantly delved into conceptual histories of psychology and psychiatry. He wrote a conceptual history of anxiety disorders, where traditional and modern understandings covering the views of B. A. Morel, M. Krishaber, Axenfeld, and S. Freud were summarized (Berrios, 1999). He also published papers discussing concepts such as “dementia” (Berrios, 1987) and “erotomania” (Berrios and Kennedy, 2002). Berrios's contributions are highly valued by numerous like-minded specialists in psychiatry all over the world (Marková and Chen, 2020).

Simultaneously, in the past decades, the eastward dissemination of modern medical knowledge since the 19^th^ century has become a subject of widespread interest. Efforts have been made to observe how terminologies, concepts, and discourses, alongside such knowledge, were introduced to China. Within this context, concepts of diseases became subjects arousing heated discussion. For instance, the concept of “diabetes” and its transmission to China have been scrutinized within the framework of “transcultural conceptual history,” as a case study on how Western medical concepts were translated, popularized, politicized, and derivatized in modern China (Miao, 2023). Nevertheless, mental disorders remain an area of insufficient research. Among the mental illnesses that have garnered significant attention over the past decades, neurasthenia stands out as one of the most extensively studied (Kleiman, 1982; Chen, 2003; Pi, 2017; Wang, 2016). Melancholia, or depression, has also received considerable attention. For example, Bai, Li, and Zhang delved into the introduction of depression in China, meticulously tracing its evolution from a symptom to an independent disease, as well as its causes from “stagnation of liver qi” to “brain dysfunction,” and examining its cultural imagery associated with femininity, the intellectual youth, and modern urban life during the late Qing and early Republican China (Bai et al., 2022).

Intriguingly, a key concept closely related to neurasthenia and melancholia as anxiety is, its introduction to the Chinese soil has not received adequate attention. Encompassing a wide spectrum of conditions, anxiety disorders include separation anxiety disorder, selective mutism, specific phobia, social anxiety disorder, panic disorder, agoraphobia, Generalized Anxiety Disorder (GAD), substance/medication-induced anxiety disorder, and anxiety disorder due to another medical condition (American Psychiatric Association, 2013). These disorders are globally recognized as some of the most prevalent and economically burdensome psychiatric conditions (Anderson and Shivakumar, 2013). Anxiety, typically characterized as an “abnormal and overwhelming sense of apprehension and fear,”^1^ has undergone significant semantic shifts over the past decades. In Freud's psychoanalytic theory, the concept of “angst” (or anxiety) plays a crucial role in understanding the development and functioning of human personality. Freud divides anxiety into three types: Realistic Anxiety, Neurotic Anxiety, and Moral Anxiety (Freud, 1943). Nonetheless, when “anxiety” became gradually recognized as a diagnostic category in the 1980s, these distinctions changed. In the ninth edition of the International Classification of Diseases (ICD-9) published in 1977, the notion of “anxiety states” was grouped with hysteria, phobic state, obsessive-compulsive disorders (OCD), neurotic depression, neurasthenia, depersonalization syndrome, and hypochondriasis as a sub-type of neurotic disorder (World Health Organization, 1977).

In the third edition of the Diagnostic and Statistical Manual of Mental Disorders (DSM-III), which was issued in 1980, GAD was formally recognized as a distinct diagnostic category (American Psychiatric Association, 1980). Subsequently, in ICD-10 released in 1992, anxiety disorders were categorized under the broader label of “neurotic, stress-related and somatoform disorders,” which covered phobic anxiety disorders, other anxiety disorders, OCD, and reaction to severe stress, adjustment disorders (World Health Organization, 1993). In 1994, DSM-IV was issued, and the notion of anxiety was further divided into a series of disorders, including panic disorder, agoraphobia, social phobia, specific phobias, OCD, posttraumatic stress disorder (PTSD), acute stress disorder, and GAD (American Psychiatric Association, 1994). In 2013, DSM-5 was issued, and further changes concerning the categories of anxiety were made, as separation anxiety disorder and selective mutism became new categories of anxiety disorders, whereas OCD and PTSD were no longer categories of anxiety disorders; the former became a category under Obsessive-Compulsive and Related Disorders, and the latter became a category under Trauma- and Stressor-Related Disorders (American Psychiatric Association, 2013). What can be seen was that the forms of anxiety disorders gradually became subdivided.

Notably, further specification can be observed in ICD-11 issued in 2018, where “Anxiety or fear-related disorders” emerged as a general category, with GAD, panic disorder, agoraphobia, specific phobia, social anxiety phobia, and separation anxiety disorder, among others, becoming its sub-categories (World Health Organization, 2019/2021). Throughout these revisions, the semantic connotations associated with “anxiety” as a mental disorder have remained in a state of continual evolution. The introduction of “anxiety,” as a notion experiencing constant semantic shifts in the Western world, thus becomes a topic worthy of further attention. It is also noteworthy that the categorization of anxiety disorders started significantly later compared to the subdivision of forms of depressive disorders, which could possibly be caused by Freud's treatment of anxiety as only “general current coin for which all the affects are exchanged, or can be exchanged, when the corresponding ideational content is under repression” (Freud, 1943). What can be felt here was that in Freud's sense, though anxiety was “not so simple a matter” (Freud, 1989), the concept itself was not considered to be as interesting as other affective states, such as depression. The incongruence between anxiety and depression in their respective courses of semantic evolution makes it also necessary to compare their introductory processes to China.

This article aims at reviewing the introduction of “anxiety” to China since the 19^th^ century. Lexicons, newspaper and periodical articles, and translated medical books were inspected and analyzed to answer the central question: how was anxiety introduced to China? Specifically, the entries related to anxiety and its associated terms were observed for a systematic and detailed account of how anxiety was translated and lexicalized in late Qing and Republican China; simultaneously, the articles on anxiety published in newspapers and journals, along with translated medical books disseminating Western psychiatric knowledge, were studied for an illustration of how anxiety was introduced and elucidated for the Chinese readers during the first half of the 20^th^ century. What follows is a section comparing the notions of anxiety in Traditional Chinese Medicine (TCM) and Western psychoanalytic approaches. After that, the introduction processes of depression and anxiety to China are compared, and the highlights of this article are summarized.

## Materials and methods

This article studied the introduction of “anxiety” to China within a thematic historical framework, and Reinhart Koselleck's criterion for conducting research of conceptual histories was adopted to select the textual materials for analysis. According to Koselleck, three types of source materials could be utilized: sources containing several temporal layers, such as lexicons and dictionaries, sources consisting of one single temporal layer, such as newspapers and letters, and the “so-called classic texts” (Koselleck, 2014). Accordingly, three types of textual evidence were employed for meticulous inspection and detailed analysis of “anxiety” in the modern Chinese context, and then its introduction to the Chinese soil was outlined.

First, the lexicographical works disseminated in China since the first half of the 19^th^ century were utilized. This type of textual evidence could be divided into two groups, the medical and non-medical lexicons, and was exhaustively selected from three different sources: “The English-Chinese Dictionary Database” in “Modern History DataBases,” “Minguo Shiqi Wenxian (民国时期文献)” of National Library of China (hereinafter referred to as “MGSQWX”), and a personal collection of medical lexicons published during 1908 and 1990 of the current author. All of the lexicons available from these three sources were carefully read and studied to outline how “anxiety” and related notions were translated into Chinese.

Specifically, the 19^th^-century English-Chinese dictionaries were scrutinized to ascertain the initial dissemination of the term “anxiety” to the Chinese populace. Medical and non-medical lexicons published subsequently in the first half of the 20^th^ century were inspected to demonstrate divergent interpretations and conceptualizations of “anxiety” that emerged during that era. Furthermore, lexicon entries after 1949 were examined to show cognition of “anxiety” as mental disorders in China became gradually understood in alignment with international diagnostic standards.

Second, the newspaper and periodical articles were selected from CNBKSY (全国报刊索引), a database maintained by Shanghai Library which contains a wide range of newspapers and periodicals published in China since 1833. Using “jiaolyu” as the search term and “title” as the search field, a search in this database yielded 100 documents published during 1833 and 1949, and through further screening, two articles introducing the concept of “jiaolyu” to Chinese readers were identified. Both articles were published in *The West Wind Monthly* (*Xi Fen* 西风), a journal founded in Shanghai in 1937, and they were meticulously analyzed to discern how “anxiety” was explained to non-professional readers.

Third, a collection of medical books published since the early 20^th^ century were studied. These books, selected from MGSQWX and a personal collection of medical publications by the China Medical Missionary Association (CMMA) (中国博医会) of the current author, were either published by CMMA, one of the most influential medical organizations disseminating Western medical knowledge in China in the first half of the 20^th^ century, or translated from Freud's writings. These materials, as the earliest textual evidence reflecting the introduction of psychiatric concepts and Freud's work to Chinese readers, helped delineate how terminologies of psychology and psychiatry evolved, as relevant knowledge was progressively and systematically introduced.

To recapitulate, the entries in the dictionaries of different types, which were related to “anxiety” and its associated terms, were carefully observed for a systematic and detailed account of how “anxiety” was translated and lexicalized in late Qing and Republican China; simultaneously, the articles on anxiety published in newspapers and journals, along with translated medical books disseminating Western psychological and psychiatric knowledge, were studied for an illustration of how anxiety was introduced and elucidated for the Chinese readers in the modern times.

## Translating anxiety: a historical journey through Chinese lexicons

An examination of English-Chinese dictionaries reveals an early inclusion of the term “anxiety.” Notably, Samuel Wells Williams' compilation, *An English and Chinese Vocabulary, in the Court Dialect*, provides an initial glimpse into this lexical entry. In this work, anxiety is rendered as “youlyu (忧虑)” (Williams, 1844). This translation could be found in a series of English-Chinese dictionaries, spanning from the mid-19^th^ century to the early 20^th^ century. For instance, in Walter Henry Medhurst's *English and Chinese Dictionary*, anxiety was also translated into “youlyu” (Medhurst, 1847–48). Beyond “youlyu,” these dictionaries offer a plethora of alternative translations, among which the first three are guayi (挂意), guaxin (挂心), and jiaosi (焦思). It can be seen that the Chinese character jiao, present in “jiaolyu zheng (焦虑症),” the contemporary Chinese translation of anxiety, has been adopted in an option of its translation since the mid-19^th^ century.

**Figure 1:**
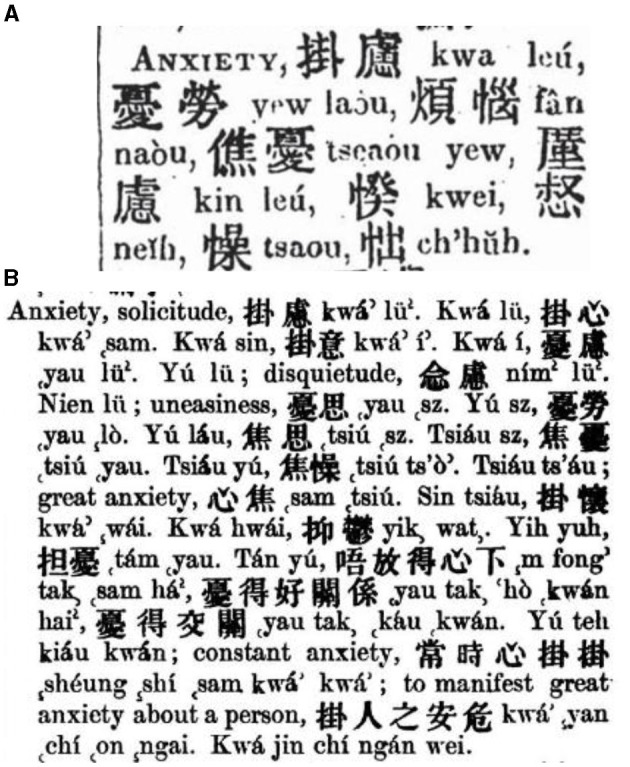
**(A)** Translations of anxiety in Medhurst's dictionary. **(B)** Translations of anxiety in Lobscheid's dictionary. Source: “The English—Chinese Dictionary Database” in “Modern History DataBases” (Institute of Modern History, Academia Sinica). Reproduced with permission from Institute of Modern History, Academia Sinica.

From then on, “youlyu” has been a translation of anxiety in Wilhelm Lobscheid's *English and Chinese Dictionary with the Punti and Mandarin Pronunciation*, Justus Doolittle's *Vocabulary and Hand-book of the Chinese Language*, Kwang Ki-chaou's *English and Chinese Dictionary*, Yen Wei-Ching's *English and Chinese Standard Dictionary*, and Karl Ernst Georg Hemeling's *English-Chinese Dictionary of the Standard Chinese Spoken Language and Handbook for Translators*, while it is noteworthy that “youlyu” was never prioritized as the primary choice for translating anxiety. Instead, alternative translations such as gualyu (挂虑) and guaxin were commonly employed. The numbers of its translations in Medhurst's (Figure 1A) and Lobscheid's (Figure 1B) lexicons manifest the difficulties in determining the translation of this concept. Without exception, all of these lexicons consistently introduced anxiety as a non-medical concept.

“Anxiety” was rendered differently in medical lexicons in the early 20^th^ century. In 1908, the first edition of *An English-Chinese Lexicon of Medical Terms* compiled by Philip B. Cousland was released. This medical lexicon, as one of the most influential English-Chinese medical lexicons in modern Chinese history, was revised over ten times during its initial release in 1908 and 1949, when its tenth edition was issued. Subsequent editions continued to appear after the founding of the People's Republic of China. In its first edition, the entry of “anxietas” was listed, and it was translated into “jiju (悸惧)” (Cousland, 1908). In Chinese, the character “ji” basically describes the heart beating with terror, and “ju” fear; therefore, “jiju” aptly captures the essence of fearfulness. This translation, along with the entry of anxietas, persisted across all editions of the lexicon published in the first half of the 20^th^ century, whereas “jiaolyu (焦虑),” the equivalent to “anxiety” in a non-medical sense in contemporary Chinese, became added as a second translation since the fifth edition released in 1924 (Cousland, 1924).

In the sixth edition of *An English-Chinese Lexicon of Medical Terms* co-authored by Cousland and Leo Teh-ching, “anxiety state” was added as an entry under anxietas. “Anxiety state” was translated into “jiaolyu qingtai (焦虑情态)” in both the sixth and the seventh editions (Cousland and Leo, 1930; Cousland et al., 1931). Since the seventh edition, the lexicon became entitled *Cousland's English-Chinese Medical Lexicon*, in memorial of Cousland, who passed away in Scotland in 1931. In its ninth edition published in 1939, three more entries of anxiety were added: “anxiety hysteria,” translated into “kongbu xing yibing (恐怖性癔病), kongbu xing xieshi tuoli (恐怖性协识脱离),” “anxiety neurosis,” translated into “jiaolyu xing shenjing (guanneng) bing (焦虑性神经[官能]病),” and “anxiety psychosis,” translated into “kongbu xing jingshen bing (恐怖性精神病), jiju xing jingshen bing (悸惧性精神病), and jiaolyu xing jingshen bing (焦虑性精神病).” Furthermore, in the same edition, the translation for “anxiety states” was refined into “jiaolyu zhuangtai (焦虑状态)” (Leo and McAll, 1939).

“Anxiety hysteria,” “anxiety neurosis,” “anxiety psychosis,” and “anxiety states” retained their presence in the eleventh edition of *Cousland's English-Chinese Medical Lexicon*, while the translations of some of these terms were simplified. “Anxiety hysteria” became “kongbu xing yibing,” and “anxiety psychosis” “kongbu xing jingshen bing” (Leo, 1953). It can be observed that since the mid-1920s the entries related to anxiety and their translations have undergone considerable changes. The 1920s was also the period when the translations of Western medical terminology were rigorously reviewed and unified by the General Committee for Scientific Terminology. The results concerning psychological and psychiatric terms were consolidated in *Pathological Terms in Psychiatry (Jingshen Bingli Xue Mingci* 精神病理学名词*)*, a 1937 publication compiled by the National Institute for Compilation and Translation and published by the Commercial Press under the guidance of the Education Commission of the Republic of China.

**Figure 2:**
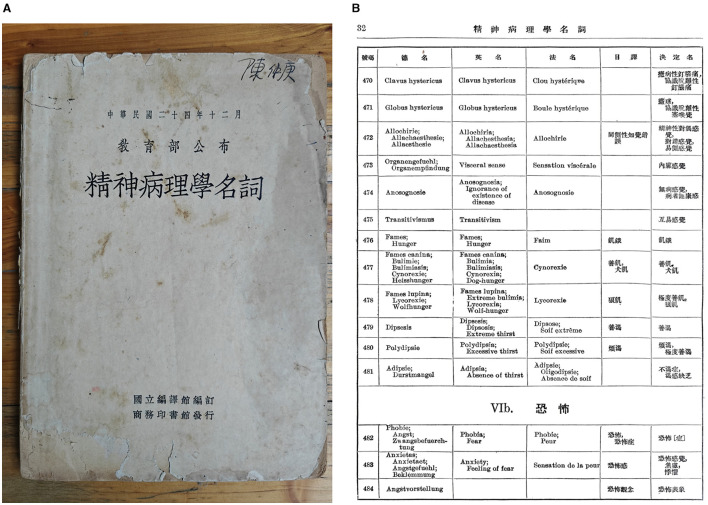
**(A)** The cover of *Pathological Terms in Psychiatry*. **(B)** The page containing “phobia” and “anxiety” in *Pathological Terms in Psychiatry* (National Institute for Compilation and Translation, 1937).

In this publication, the German term, English term, French term, Japanese translation, and determined Chinese translation of 1,173 entries are provided. The 482^nd^ entry is “Phobia, Fear,” of which the German terms include “Phobie, Angst, Zwangsbefuerchtung,” and the determined term is “kongbu zheng (恐怖[症]).” The 483^rd^ entry is “Anxiety, Feeling of fear,” of which the German terms include “Anxietas, Anxietaet, Angstgefuehl, Beklemmung,” and the determined term are “kongbu ganjue (恐怖感觉)” (which literally means a feeling of fear), “jiaolyu,” and “jiju” (National Institute for Compilation and Translation, 1937). Notably, both translations of “anxietas” provided by *Cousland's English-Chinese Medical Lexicon* were adopted by the National Institute for Compilation and Translation as valid renderings for “anxiety,” while neither was accorded primary status. These two entries were both grouped within the broader category of “Phobia” (Figure 2), where most sub-categories pertained to specific phobias. Two exceptions were “Examination anxiety” and “Stage-fright.” In this context, “anxiety” can indeed be viewed as a synonym for “phobia.”

Based on meticulous inspection of the entries related to anxiety in medical lexicons and *Pathological Terms in Psychiatry*, a tentative conclusion emerges: the translation of anxiety was not standardized before 1940. For one thing, anxiety was not acknowledged independently as a medical term; instead, “anxietas,” the German term for anxiety, persistently appeared in *Cousland's English-Chinese Medical Lexicon*. For the other, the relation between anxiety and phobia remained unclear. Nonetheless, “jiaolyu” was visible among the entries associated with anxiety, which prepared the Chinese readers for the reception of this concept coming from Western psychiatry.

An understanding of the phrase “jiaolyu” can be achieved through an analysis of the characters “jiao” and “lyu.” According to *An Explanation of Written Characters* (*Shuo Wen Jie Zi*, 说文解字), “jiao” primarily means “being hurt by fire”; “lyu” means “deliberate and considerate thought” (Xu, 1978). In the Chinese language, the image of fire is usually associated with a sense of hurry and worry. Thus, “jiaolyu” literally reflects a state of being overwhelmed by haste and overburdened with thoughts. Etymologically, “jiaolyu” is obviously different from “anxietas,” the latter originating from the Latin root “*ango*” (or “*anxi*”), which means “to strain, press, strangle, choke, vex, or trouble” (Wedgwood, 1872).

## Deciphering anxiety: insights from newspapers, journals, and translated medical books

The entries associated with anxiety in lexicons spanning the mid-19^th^ century to the mid-20^th^ century reflect both the medical and the non-medical facets of the concept. Likewise, publications in the early 20^th^ century introduced Western medical knowledge through both professional and non-professional channels. In that period, newspaper and periodical articles disseminating information on concepts and theories of Western medicine were published, and books about Western medical knowledge were translated into Chinese. Generally speaking, such knowledge was initially introduced to Chinese readers via newspaper and journal articles. For example, in the case of diabetes being introduced to the Chinese public, the earliest efforts made to promote relevant knowledge were documented by publications like *Yi Yao Xue Bao* (医药学报), *Hua An* (华安), *Shi Bao* (时报), and *Qi Lu Yi Kan* (齐鲁医刊). Subsequently, diabetes knowledge became systematically explained in books, such as Diabetes by Bertram and Jiang (1936).

The case of anxiety could be regarded as an exception, as newspaper and periodical articles rarely delved into it as a medical concept. After all, anxiety was not officially acknowledged as a mental disorder by Western medicine at that time. Articles introducing anxiety as a disorder, or as a cause for disorders, could be found in *The West Wind Monthly*, which aimed at translating and telling the essence from Western periodicals and introducing life and society in Europe and America. An article in this journal published in 1939 entitled “Anxiety Causing Diseases (Jiaolyu Zhiji 焦虑致疾)” introduced the belief that fannao (worries 烦恼) was a direct cause of unhealthy bodily conditions. The article was a translation of George W. Gray's article by Fang Ren (放任). It begins with a story of a man suffering from eczema, a result of his constant worries about when his young fiancé would make a decision on the date of their marriage. Such examples are used by the author to illustrate that some illnesses can be explained by emotional reasons, and that fear and anger might lead to significant bodily changes (Fang, 1939).

Another article introducing anxiety as a disease concept in *The West Wind Monthly* was entitled “Jiaolyu.” It was authored by Ding Zan (丁瓒) and published in 1948. This article introduces the up-to-date psychological research at that time. In the beginning, the author draws the readers' attention to the issue of terminology concerning psychological disorders. In this context, two types of psychological disorders are introduced: jingshen bing (psychoses 精神病) and jingshen shenjing bing (psychoneuroses 精神神经病), or more commonly speaking, guanneng shenjing bing (neuroses 官能神经病). The latter, which was prevailing in society, could be further divided into four sub-types: xie si die li ya (hysteria 歇斯迭里亚), jingshen shuairuo (psychasthenia 精神衰弱) (which includes phobias and obsessions and compulsion), shenjing shuairuo, and jiaolyu zheng (anxiety). The author adds that “jiaolyu zhuangtai” (“anxiety state” in the brackets) is the most basic phenomenon concerning psychological disorders, and it is related to but different from “kongju” (“fear” in the brackets) (Ding, 1948).

The abovementioned articles in *The West Wind Monthly* both made initial efforts to inform the Chinese public of the belief in Western psychology that “anxiety” was a psychological disorder, which could cause unhealthy bodily conditions. Nevertheless, these articles were not the earliest texts disseminating knowledge on mental disorders in the Chinese psychological domain. Early in 1913, a book entitled *Ling Xin Bing Jian Shu* (灵心病简述) was published by the Publication Committee of CMMA. It was a translation from E. G. Younger's *Insanity in Every-day Practice* by Maison J. Chu (朱剑) and Cousland. This book could be regarded as one of the earliest publications introducing mental disorders to the Chinese people. In this first edition, “insanity” was translated into “dianzheng 癫症,” and the different types of insanity, such as mania (kuangzheng 狂症), melancholia (aizheng 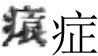, yizheng 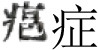), and dementia (jiezheng 㾏症), and the special forms of insanity were respectively introduced (Younger et al., 1913).

In 1929, the second Chinese edition of *Ling Xin Bing Jian Shu, Jing Shen Bing Jian Shu (*精神病简述*)*, which was translated from the fifth edition of *Insanity in Every-day Practice*, was published by the Council on Publication of China Medical Association. As was clarified in Preface to the Second Edition, in this new edition, the terminology of psychiatry had been updated by Leo The-ching and Cousland. Indeed, the translations of considerable terms became different from those in the first edition. For example, “insanity” was translated into “jingshen cuoluan (精神错乱),” “mania” into “zaokuang (躁狂),” “melancholia” into “youyu bing (忧郁病),” and “dementia” into “chidai (痴呆)” (Younger et al., 1929). Many of these translations were becoming close to the unified terms in the Chinese language today. Regarding the fact that “anxiety” was not classified as a mental disorder by Western psychiatry at that time, it was not mentioned in either edition.

The concept of “anxiety” was not introduced as a psychiatric one until the publication of *Jingshen Fenxi Yinlun (A General Introduction to Psychoanalysis* 精神分析引论*)*, a translation by Gao Juefu (高觉敷) from *A General Introduction to Psychoanalysis* translated by Joan Riviere. In this early work acquainting the Chinese readers with Freudian psychoanalytic terminology, a series of key notions were translated and introduced, such as “psychology of errors” (guoshi xinli xue 过失心理学), “neuroses” (shenjing bing 神经病), “psychoanalysis” (jingshen fenxi fa 精神分析法), “psychiatry” (jingshen liaobing fa 精神疗病法), “Libido” (jili 基力), “nervousness” (shenjing guomin xing 神经过敏性), and “anxiety” (jiaoji 焦急). The 25th lecture in the book was entitled “jiaoji,” where Freud explained and discussed different types of nervous anxieties.

The psychoanalyst differentiated between anxiety, fear, and fright by arguing that “anxiety relates to the condition and ignores the object, whereas in the word fear attention is directed to the object; fright does actually seem to possess a special meaning—namely, it related specifically to the condition induced when danger is unexpectedly encountered without previous anxious readiness.” In these lines, “fear” was translated into “jingju 惊惧,” and “fright” into “jinghai 惊骇.” The three types of neurotic anxieties (shenjing bing de jiaoji 神经病的焦急), according to Freud, were (1) a “free-floating” anxiety (piaofu zhe de jiaoji 飘浮着的焦急); (2) the anxiety of the extraordinary various and often very peculiar phobias (jingji bing 惊悸病); (3) an entirely unintelligible group featured by “anxiety equivalents” (Freud and Gao, 1930). In this context, the notion of “anxiety” was, for the first time, conceptualized as a mental disorder in the Chinese language.

The Chinese term “jiaoji” emphasizes the state of being hurried, whereas “jiaolyu” emphasizes the sense of worry and fear. As such, “jiaoji” has a greater distance from “anxiety” in modern psychiatry, and thus the phrase was no longer adopted as a technical term in psychology or psychiatry after the founding of the People's Republic of China.

## Anxiety in Chinese discourse after 1949

Xu et al. meticulously encapsulate the evolution of epidemiological research on mental disorders in China after 1949, discerning three distinct periods in this development. The initial period, spanning from 1958 to 1981, was characterized by the reliance on the 14-category classification standard, which was established at the First National Conference on Mental Disease Prevention and Control held in Nanjing in 1958. The second period, extending from 1982 to 2000, witnessed a significant shift in diagnostic paradigms with the integration of international classification systems, including ICD-9, DSM-III, and CCMD. The third and current period, commencing in 2001, has been distinguished by the adoption of the most recent iterations of ICD and DSM as diagnostic criteria; this period has also seen the introduction of complex sampling techniques (Xu et al., 2015).

During the initial and second periods of mental health epidemiology in China, the concept of anxiety did not feature prominently in Western psychiatric discourse as a distinct medical construct. As a result, discussions pertaining to anxiety were relatively constrained. The symptoms associated with anxiety, as well as those indicative of other mental health conditions, were often subsumed under the umbrella term of neurasthenia. These symptoms included appetite changes, sleep disturbances, and weakness or fatigue. Consequently, the morbidity of neurasthenia exceeded 80% in the Chinese psychiatric outpatients' departments in the 1950s (Lin, 1992). Even 30 years later, in the 1980s, the morbidity of neurasthenia was still 13.03‰, accounting for 58.7% of all neurosis cases (He, 2017).

Within this framework, the scope for scholarly research and further discussions on “anxiety” was markedly restricted. Despite these constraints, the term “anxiety” (along with “anxietas”) managed to find its way into the lexicon of mainstream medical dictionaries. The equivalence between “anxiety” and “jiaolyu” (as well as “jaolyu zheng”) was thus fixed during this period.

A significant event regarding Chinese medical terminology after 1949 was the publication of *Dr. Chao's New Medical Lexicon* (赵氏英汉医学辞典) in 1952 by Chung Wha Book Company (中华书局). In this dictionary including over ninety thousand entries, “anxietas” and “anxiety” were both listed, the former explained as “Latin for anxiety” and translated into “jiju, jiaolyu, kongbu 恐怖,” and the latter also translated into “jiju, jiaolyu, kongbu.” Two terms, anxiety neurosis (jiju xing shenjing bing 悸惧性神经病, jiaolyu xing shenjing bing 焦虑性神经病) and anxiety psychosis (jiju xing jingshen bing 悸惧性精神病, jiaolyu xing jingshen bing 焦虑性精神病) were listed under the entry of anxiety (Zhao, 1952).

**Figure 3:**
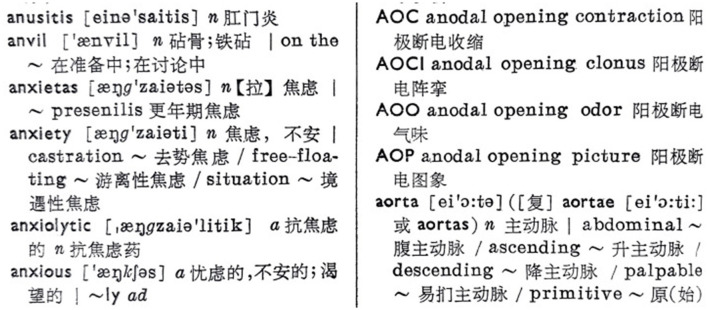
A screenshot of the page demonstrating the entries concerning anxiety in *An English-Chinese Medical Dictionary* (Chen et al., 1984). Source: a hard copy of *An English-Chinese Medical Dictionary* owned by the author. Reproduced with permission from Shanghai Scientific and Technical Publishers.

Five years later, a new edition of *Yixue Mingci Huibian* was published by People's Medical Publishing House. In this lexicon, “jiaolyu” became the only translation of the entry “anxietas; anxiety” (People's Medical Publishing House, 1957). Since then, the inclusion of “jiaolyu” as a translation of “anxiety” became steadfast, and in most cases, “jiaolyu” was the first choice. For example, in both *English-Chinese Glossary of Basic Medical Terms* published in 1976 and *Yinghan Yixue Cihui (English-Chinese Medical Terminology* 英汉医学词汇*)* published in 1978, “anxiety” was translated into “jiaolyu,” and “anxiety situation” into “xin qing kuang jiaolyu (新情况焦虑)” (People's Medical Publishing House, 1976, 1978). In *An English-Chinese Medical Dictionary* published in the 1980s, “jiaolyu” was the first choice for both “anxiety” and “anxietas” (Chen et al., 1984) (Figure 3).

## Anxiety in TCM and western psychoanalytic approaches

Although modern psychology and psychiatry were not transmitted to China until the turn of the 20^th^ century, the concept of “anxiety” could be observed in classical medical books of China. In *The Yellow Emperor's Inner Classic: Plain Conversation* (*Huang Di Nei Jing: Su Wen*, 黄帝内经*:* 素问, hereinafter abbreviated as “HDNJSW”), a chapter entitled “Discussion on Wind” (Feng Lun, 风论) is devoted to explaining “feng xie 风邪,” a type of exogenous pathogen related to the wind, as a category of cause for diseases (Yao, 2022). In TCM, the exogenous pathogens are labeled as “xieqi 邪气,” of which “six excesses,” “pestilential qi,” “excess of seven emotions,” “dietary irregularities,” “lack of exercises or excessive labor,” and “insect bites or animal injuries” are common categories (Li et al., 2021).

Within this chapter, the notion of “xin feng 心风,” a type of disease of the heart in TCM theories, is introduced to the readers, and its symptoms including malfunctioning of sudoriferous glands, emaciation, and constant anger are explained (Yao, 2022). Interestingly, it can be observed that mental disorders in ancient Chinese classics are often related to the heart rather than the brain. This stemmed from the conventional belief that the heart served as the governing force of the body. As is explained in “Discussion on the Secret Canons Stored in Royal Library” (Linglan Midian Lun, 灵兰秘典论), another chapter in HDNJSW, the heart is the “monarch” (junzhu zhi guan, 君主之官), who generates wisdom (Yao, 2022). In *Essentials of Internal Classic* (*Nei Jing Zhi Yao*, 内经知要), a classical work facilitating people's understanding of the basic theories of TCM in the Ming Dynasty (1368–1644), the heart is further explained as “the governor of the whole body” (yishen zhi zhu, 一身之主) (Li, 2007).

Interestingly, such an understanding of the heart resonated with the view of Democritus, one of Hippocrates' contemporaries, that the heart was the “queen” of the body, functioning as the controller of the emotions (Mavrodi and Paraskevas, 2014). Similar views were held both by his predecessors, such as Empedocles in the pre-Hippocratic era, who believed that the heart was the most essential organ due to its role in distributing “life” via the blood vessels (Mavrodi and Paraskevas, 2014), and by his descendants, such as Galen, who claimed that rationality was seated in the brain, while emotions were thought to originate either throughout the entire body or from some specific viscera, such as the heart and the liver (Freemon, 1994). This classic Galenic view on the body and mental processes persisted in the 18^th^ century. Even in the late 19^th^ century, the connection between “xin feng” and anxiety built through the notion of the heart was still observable. In the 1870s, Da Costa observed over 300 patients and introduced the concept of “an irritable heart,” which was subsequently referred to as “soldier's heart” and “heart neurosis.” Such a connection continued to exist even after 1950 (Glas, 2003).

Nonetheless, the Chinese and Western medicines' views on the controller of the body began to differ in the last decade of the 18^th^ century, when the mysteries of brain organization were gradually unraveled, with Franz Gall being the first physician to explain the mind-brain relation by identifying the cerebral cortex as the primary region housing the organs that support intellectual functions (Simpson, 2005). It is manifest that the traditional Chinese perspective on the “monarch” or “governor” of the body is largely different from the modern one that the body is governed by the brain, a belief introduced to China in the 19^th^ century, when Western anatomical knowledge gradually disseminated in this country, first by Benjamin Hobson in his famous translation *New Treatise on Physiology* (*Quanti Xinlun*, 全体新论) published in 1851, and recognition shifted toward brain as the central locus of human emotions (Chang, 2011).

Indeed, the modern psychoanalytic view on anxiety and the Chinese folk conception of it differ intrinsically. The anxiety disorders underwent a process of classification, during which “anxiety neurosis” was the first to be recognized as a distinct stratum of neurasthenia, and various forms of it were then gradually classified (Glas, 2003); however, the concept of “xin feng” was abstract and experiential. The symptoms of “xin feng” recorded by HDNJSW could hardly make it an independent notion of disorder within psychoanalysis; therefore, the concept could neither be understood as a sub-type of a mental condition nor be accepted as a hypernym of different mental disorders. Given such a feature of “xin feng,” anxiety, throughout its process of introduction to China, has always been accompanied by an abstract and general sense, which could be observed in its translations (mainly in the form of Chinese terms for daily and non-medical mental states).

## Discussion

An analysis of entries related to “anxiety” in English-Chinese lexicons published during the mid-19^th^ century and the early 20^th^ century helps reveal diverse translations of this concept, including “youlyu,” “gualyu,” “guayi,” “guaxin,” and “jiaosi,” none of which demonstrates its status as a formal medical construct. Subsequently, in the early 20^th^ century, “anxiety” became recognized as a disease concept in medical lexicons, where “jiju” and “jiaolyu” became its common equivalents. Concurrently, a series of terms, including “anxiety state,” “anxiety hysteria,” “anxiety neurosis,” and “anxiety psychosis,” were translated and explained. The entries pertaining to “anxiety” in medical lexicons in that period help demonstrate that the standardized translation of “anxiety” was not produced before 1940. Its translation in contemporary Chinese, “jiaolyu,” became increasingly standardized in medical lexicons after 1949.

As “anxiety” became a common entry in English-Chinese dictionaries of different types, it was also mentioned and explained in newspaper and periodical articles before the mid-20^th^ century. Notably, the introduction of “anxiety” by these articles is dual-faceted; the feature of the concept as a mental disorder became recognized by the authors around 1940. Synchronically, in these articles, core terms, such as psychosis, psychoneurosis, hysteria, and psychasthenia, became translated and introduced. Likewise, psychological and psychiatric terminologies were introduced, and updated in medical books translated and published since the early 20^th^ century, where the evolution of these terminologies in the Chinese language before the founding of the People's Republic of China could be traced.

The translation of terms concerning anxiety and related disorders did not conceal the fact that “anxiety” was not recognized as a legitimate medical construct before the end of the 20^th^ century. In the 1980s, “neurasthenia” was still a mainstream concept in Chinese psychiatry and psychology, which was revealed by A. Kleiman's seminal research in the Psychiatry Outpatient Clinic at the Hunan Medical College in the summer of 1980. Among all patients studied by him, 15% were diagnosed with “neurosis” by psychiatrists at Hunan Medical College. The category of “neurosis” covered neurological headaches, vascular headaches, anxiety states, phobias, and OCD. Anxiety was not considered an independent category (Kleiman, 1982).

The percentage of “depression” cases, 1%, was much lower than that of “neurosis,” reflecting the situation recorded by Kleiman (1982) that “depressive disorder rates in China are either unreported or extremely low”. When DSM-III was used, among the 100 neurasthenia patients, 93 were diagnosed with depression, and 69 were diagnosed with Anxiety Disorders (Kleiman, 1982). Kleiman (1982)'s empirical study indicated that depression and anxiety, originally regarded as symptoms rather than independent medical constructs, did not become valid diagnostic categories in psychiatry immediately after their terminologies were translated into the Chinese language. The introduction processes of depression and anxiety to China could be considered similar: both were loose notions of psychiatric symptoms before the introduction of international diagnostic standards, and both gradually became legitimate psychiatric constructs when “neurasthenia” faded.

Such similarity is worthy of further attention, as an inconsistency between depression and anxiety in their respective conceptual histories could be observed. “Depression” could be found in psychiatric classifications in the late 19^th^ century (Jackson, 1986), which means it was regarded as a formal diagnostic category long before the ICD or DSM was issued, whereas the status of anxiety was in constant changes during the revision of these diagnostic standards. In this sense, the psychoanalytic approaches to depression and anxiety were different, but the processes of their introduction to China were not.

What made a difference was the precipitous decline experienced by the discourse on neurasthenia in China, as the 20^th^ century drew to a close. Concurrently, this period marked the genesis of a more nuanced and proliferative discourse on mental disorders. The retreat of neurasthenia from this discourse did not merely connote its dilution into other existing categories, such as melancholia, anxiety, and insomnia. Rather, it heralded the advent of a novel epoch of psychiatric development in China, which was characterized by an enhanced specificity in the classification and understanding of mental disorders. Such specificity was never observed in the explanation for the concept of “xin feng.”

In the course of anxiety's introduction to the Chinese culture, corresponding TCM notions were not highly involved. The TCM term “xin feng” was not transplanted into the translations of “anxiety,” and theories explaining “xin feng” as a disease caused by “feng xie” were not utilized in the promotion of knowledge concerning anxiety and related disorders. In other words, “anxiety” was introduced to China as a new concept, which may not find its root in the rich traditions of TCM, and the Chinese discourse pertaining to it has, in the past decades, undergone a process of rejuvenation, aligned with the iterative refinements of ICD and DSM.

## Conclusions

This article reviews the introduction of “anxiety” to China since the 19^th^ century by inspecting and analyzing lexicons, newspaper and periodical articles, and translated medical books, and concludes that as a term included in lexicons published in the mid-19^th^ century, “anxiety” was translated into a series of Chinese terms, and “jiaolyu,” one of its translations, gradually became its standardized translation after 1949. Simultaneously, knowledge concerning anxiety was disseminated in newspaper and periodical articles, as the notion of “anxiety” became introduced as a mental disorder. Nonetheless, “anxiety” did not become a legitimate medical construct until the end of the 20^th^ century.

Notions similar to anxiety can be found in TCM, among which “xin feng” is a typical example. Although a connection between traditional Chinese and Western views on anxiety conditions can be observed through the notions of “soldier's heart” and “heart neurosis,” “xin feng” and “anxiety” differ intrinsically, as the former is abstract and experiential, whereas the latter has undergone a process of classification until it becomes scientifically recognized as a diagnostic category. The introduction of “anxiety” to China was similar to that of “depression,” as neither was adopted efficiently as a diagnostic category in Chinese psychiatric outpatient departments before the introduction of international standards, such as ICD and DSM.

What is presented in this article is a thematic historical study on the introduction of “anxiety” to China. It is hoped that this case of “anxiety” could help reveal how Western psychiatry was translated and introduced to the Chinese public throughout history. To better understand the local Chinese conditions welcoming Western psychiatric concepts, disorders similar to depression and anxiety in TCM are to be explored and analyzed. To better understand how Western psychiatry and psychology were promoted and popularized in China, more notions within the field of psychopathology are to be studied, with their itineraries to China traced and their meaning construction within the Chinese language outlined.

## Footnotes

^1^“Anxiety.” *Merriam-Webster.com* *dictionary*, Merriam-Webster. Available at: https://www.merriam-webster.com/dictionary/anxiety (accessed March 25, 2024).
